# 

**Published:** 1893-02

**Authors:** 


### CONTENTS:

ORIGINAL ARTICLES: PAGE

Record of a Case of Glanders in Man, with History of Infection from a Horse, together with a Clinical and Post Mortem Record of the Case in the Suspected Animal. By A. W. Clement, V. S. .... 65
Seedy Toe. By Williamson Bryden, V. S. .... 69
Diseased Meats as Food. By Claude D. Morris, V. S. .... 74

SELECTIONS:

A Complicated Case of Parturition. By E. Bowman, M.R.C.V.S., Glasgow .... 79
Some Suggestions for Better Country Roads. By Sarah Cooper Hewitt .... 81
Mallein as an Aid to the Diagnosis of Glanders. By W. Hunting, F.R.C.V.S., London, and J. M'Fadyean, M.B., B.Sc., Royal Veterinary College, London .... 87
Malformation of Alimentary Canal. By Mr. A. G. Bowker .... 101

COMMUNICATIONS:

Veterinary Science and the United States Army. By R. W. Shufeldt, Med. Dept., U. S. A. .... 102
Pleuro-Pneumonia on Boston Ships. Williamson Bryden .... 105

EDITORIAL:

Public Highways .... 107

SOCIETY PROCEEDINGS:

United States Veterinary Medical Association .... 109
The New York State Veterinary Medical Society .... 112
The New York State Veterinary Medical Society .... 125
The Massachusetts Veterinary Association .... 126

